# Electrical impedance spectroscopy and tomography for characterizing electrical changes associated with amoxicillin dispersion in *ex vivo* biological tissues

**DOI:** 10.2478/joeb-2026-0018

**Published:** 2026-11-09

**Authors:** Manuel Vázquez-Nambo, José-Antonio Gutiérrez-Gnecchi, Enrique Reyes-Archundia, Wuqiang Yang, Daniel Lorias-Espinoza, Marcial Reyes-Cazares

**Affiliations:** TECNM/Instituto Tecnológico Superior de, Pátzcuaro, Michoacán, México; TECNM/Instituto Tecnológico de Morelia, División de Estudios de Posgrado E Investigación, Michoacán, México; Department of Electrical and Electronic Engineering, The University of Manchester, Manchester, UK; CINVESTAV/IPN, D. F., México

**Keywords:** Drug dispersion, bioimpedance, electrochemical impedance spectroscopy, resistivity maps, tissue conductivity

## Abstract

Studies of ionic liquid dispersion in biological samples are essential for improving dosages, minimizing side effects, and increasing treatment efficacy. Therefore, monitoring drug dispersion is crucial for understanding spatial and temporal drug transport.

**Objective:**

The study evaluates electrochemical impedance spectroscopy (EIS) and electrical impedance tomography (EIT) to characterize electrical changes associated with amoxibron administration and its spatial propagation in ex vivo tissues, in a frequency range of 1 Hz to 10 kHz for EIS and 10, 50, and 100 kHz for EIT.

**Materials and methods:**

A gold electrode was used for EIS and a 16-electrode array for EIT. Three 5-mL doses of the amoxibron (C_16_H_19_N_3_O_5_S) drug were injected into each tissue sample at 2-min intervals. The electrical changes were analyzed using impedance spectra, equivalent electrical models, and resistivity distributions. To support the study, an additional test in beef tissue was conducted that included a measurement from 0 to 22 minutes.

**Results:**

At low frequencies, beef tissue showed the highest impedance of 20 kΩ, followed by pork and chicken with values of 11 kΩ and 10 kΩ, respectively. Drug administration impedance was reduced by approximately 50% in beef, 40–45% in chicken, and 10–15% in pork. The fitting accuracies of the state-space models were above 90% in all samples.

**Conclusions:**

The resistivity maps showed diffusion resulting from impedance differences between tissue treated and untreated with the drug. Beef and chicken showed similar diffusion toward the periphery, whereas pork showed minimal changes over time toward the center. The temporal and spatial electrical changes were associated with amoxibron delivery, with differences in electrical response among tissues. The spatial resolution and localization performance of the EIT images were quantified using full width at half maximum (FWHM). The localization error was approximately 44 to 59% relative to the FWHM.

## Introduction

The study of drug dispersion in tissues plays an important role in the pharmaceutical sciences. Monitoring the propagation of medicine is essential for an effective therapy. This involves the absorption, distribution, and time required to metabolize the drug in the tissue [1]. The correct dispersion ensures that the drug reaches the lesion in effective concentrations, minimizing the side effects and the damage to surrounding cells. Parameters such as viscosity, porosity, and the impedance difference between the drug and tissue may influence the effective absorption of liquid [2, 3]. The locoregional method has been used in the last years to treat focalized lesions successfully, reducing toxicity levels and increasing therapeutic effectiveness [4, 5]. The procedure involves the direct injection of the drug into damaged tissue [6, 7]. Although drug administration is commonly considered in the context of focalized tumor microenvironments, the present study does not use tumor cells or an in vivo tumor model. Instead, ex vivo beef, chicken, and pork tissues were selected as experimental biological matrices to investigate the electrical changes associated with administration and dispersion of the drug formulation.

These tissues provide controlled experimental conditions for evaluating EIS and EIT response investigations in physiologically representative models [8, 9]. Figure 1 shows an experimental flow diagram of drug dispersion in biological tissues using EIS and EIT.

**Figure 1:**
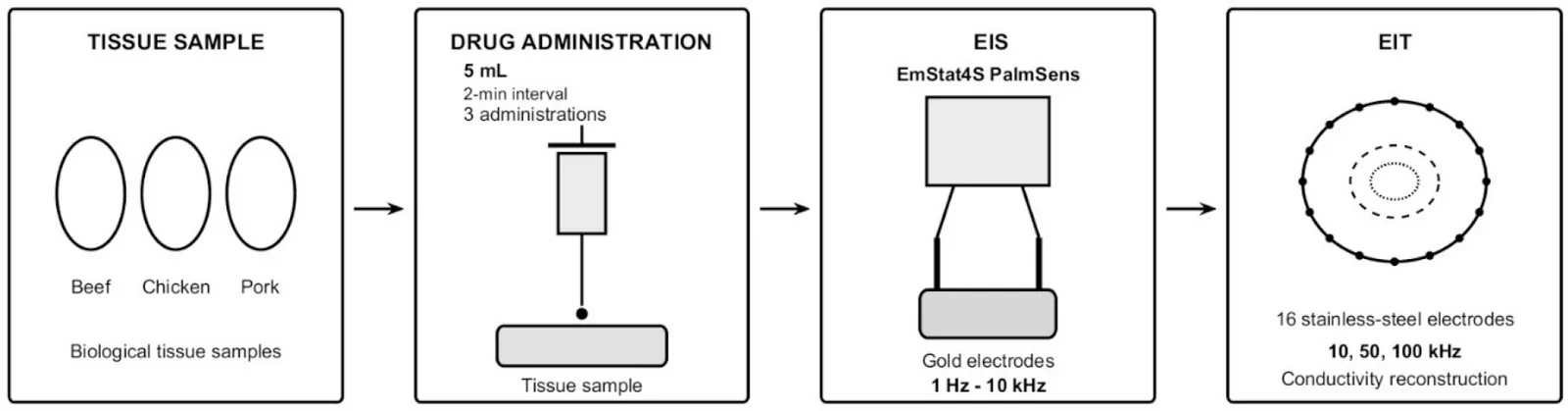
Experimental workflow for monitoring drug dispersion in biological tissues using EIS and EIT.

To study the dispersion of amoxicillin in the biological tissue, the first step was the characterization of the electrical response, using EIS over a frequency range of 1 Hz–10 kHz. In the second step, EIT measurements are then performed using a 16-electrode configuration at 10, 50, and 100 kHz to monitor spatial changes in the electrical properties of the tissue. Finally, the acquired measurements are used for conductivity reconstruction through the finite element method (FEM) to evaluate the distribution of conductivity changes associated with amoxicillin dispersion to be evaluated. [10, 11, 12], as shown in Figure 2.

**Figure 2:**
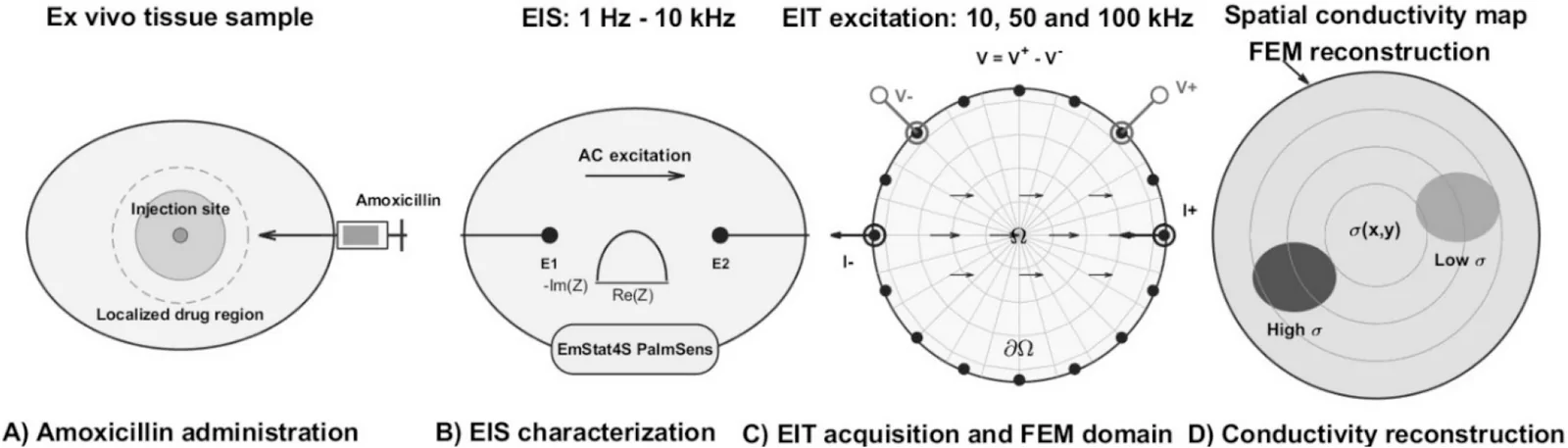
Schematic diagram of A) localized dispersion near the injection site, B) analyses with EIS, C) reconstruction of the domain using FEM, D) images reconstructed of possible transport patterns that produce spatial conductivity variations detectable by EIT.

The diffusion of drugs in tissues involves phenomena such as elevated interstitial fluid resulting from high pressures. This leads to a rapid decrease in the fluid as it disperses into the tissue. In contrast, low pressure may generate convective transport that hinders drug penetration, causing the drug concentration to decrease with distance. Even if the fluid is inside a lesion, the effectiveness of the drug decreases and increases treatment resistance (Figure 3).

**Figure 3:**
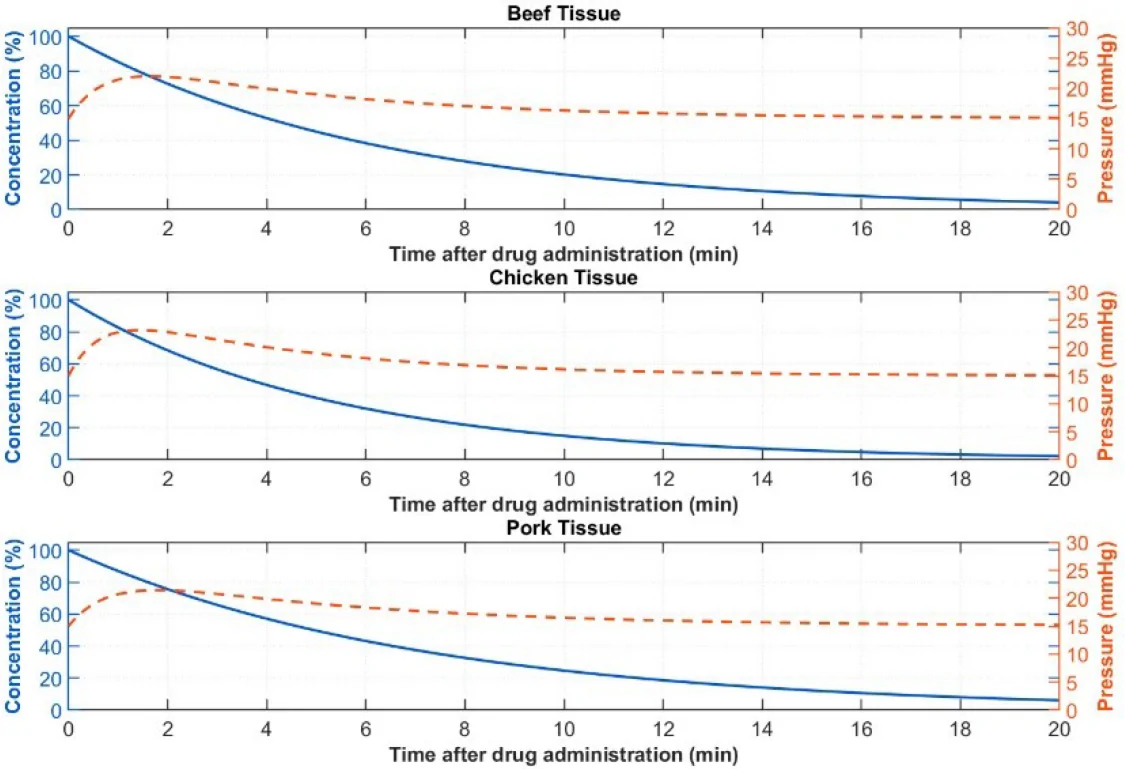
Temporal evolution of fluid pressure and drug concentration for beef, chicken, and pork tissues.

However, although the transport phenomena may produce changes in the electrical response, the impedance cannot by itself be interpreted as direct evidence of molecular drug diffusion because impedance is influenced by several factors, including ionic concentration, tissue hydration, electrode–tissue interface effects, and structural heterogeneity. Therefore, an independent reference measurement is required to establish whether the spatial conductivity changes reconstructed by EIT correspond to the actual distribution of the administered drug.

Previous studies show the potential of impedance techniques for monitoring drug transport and distribution in biological systems. Specifically, EIS is used to study and evaluate transdermal drug delivery in *ex vivo* and *in vivo* models, and EIT is used to monitor drug release and reconstruct time-varying spatial conductivity distributions. The novelty of the present study is the combined application of EIS and EIT for the characterization of the electrical changes associated with amoxicillin administration in beef, chicken and pork tissues under controlled ex vivo conditions [12, 13].

Other contributions that support the present study include work by Weiser and Saltzman, who highlighted the role of controlled-release methods for local delivery in the brain, blood vessels, mucosal epithelia, and skin [14]. Moreover, Baish *et al*. found that the delivery of blood-borne molecules and nanoparticles in the blood varies between tumor and normal tissues owing to differences in the vascular structures [15]. On the other hand, Ding *et al.* examined electrospinning as a technique for drug delivery systems with application in cancer therapy [16]. Additionally, Pertici *et al.* developed an injectable and biodegradable hydrogel intended for use in drug delivery, the brain, and soft tissue [17].

The objective of this study was to investigate the feasibility of combining EIS and EIT to characterize the temporal and spatial dispersion of amoxicillin in ex-vivo biological tissues. In particular, with emphasis on identifying impedance parameters associated with transport phenomena. EIS was used to identify the electrical signatures of the tissues and drug using impedance techniques, exploiting conductivity variations involved in bioelectrical processes characteristic of biological systems. Additionally, conductivity gradients of consecutive EIT reconstructions were analyzed to identify spatial and temporal variations in the electrical properties of the tissue following drug administration. These variations were considered as electrical indicators associated with the dispersion of the administered drug rather than direct measurements of drug concentration.

## Materials and methods

### Materials

Amoxicillin, bromhexine C_16_H_19_N_3_O_5_S (amoxibron) was used because of water solubility and molecular weight. These properties are important for drug diffusion in biological tissues and for the identification of conductivity gradients, ensuring reliable and reproducible results. The experiment array included an PalmSens EmStat4s^®^, which was used for electrochemical impedance spectroscopy measurements. Its dimensions are 7.2 × 5.5 × 2.6 cm, which include a circular gold electrode of 7.07 mm^2^ with a thickness of 650 μm and a contact pad pitch of 2.54 mm. It was configured to an excitation signal of 0.9 V in a frequency range of 1 Hz to 10 kHz. On the other hand, the EIT setup included a phantom of a radius of 12.7 cm, 16 electrodes of stainless steel with a separation of 2.44 cm between electrodes, a rectangle geometry of 2.54 × 1.27 cm, and a thickness of 0.12 cm. The reference material was tap water with a conductivity of 423 µS/cm. The procedure for data acquisition incorporated software, a LattePanda Alpha^®^ integrated into an Arduino Leonardo^®^ board, and an output multiplexer system. The temperature for all samples was 25°C, with excitation voltages of 200 mV, 400 mV, 1 V, and 2 V at frequencies of 10, 50, and 100 kHz. The array maintained a stable voltage to reduce noise and improve measurement accuracy, as shown in Figure 4.

**Figure 4:**
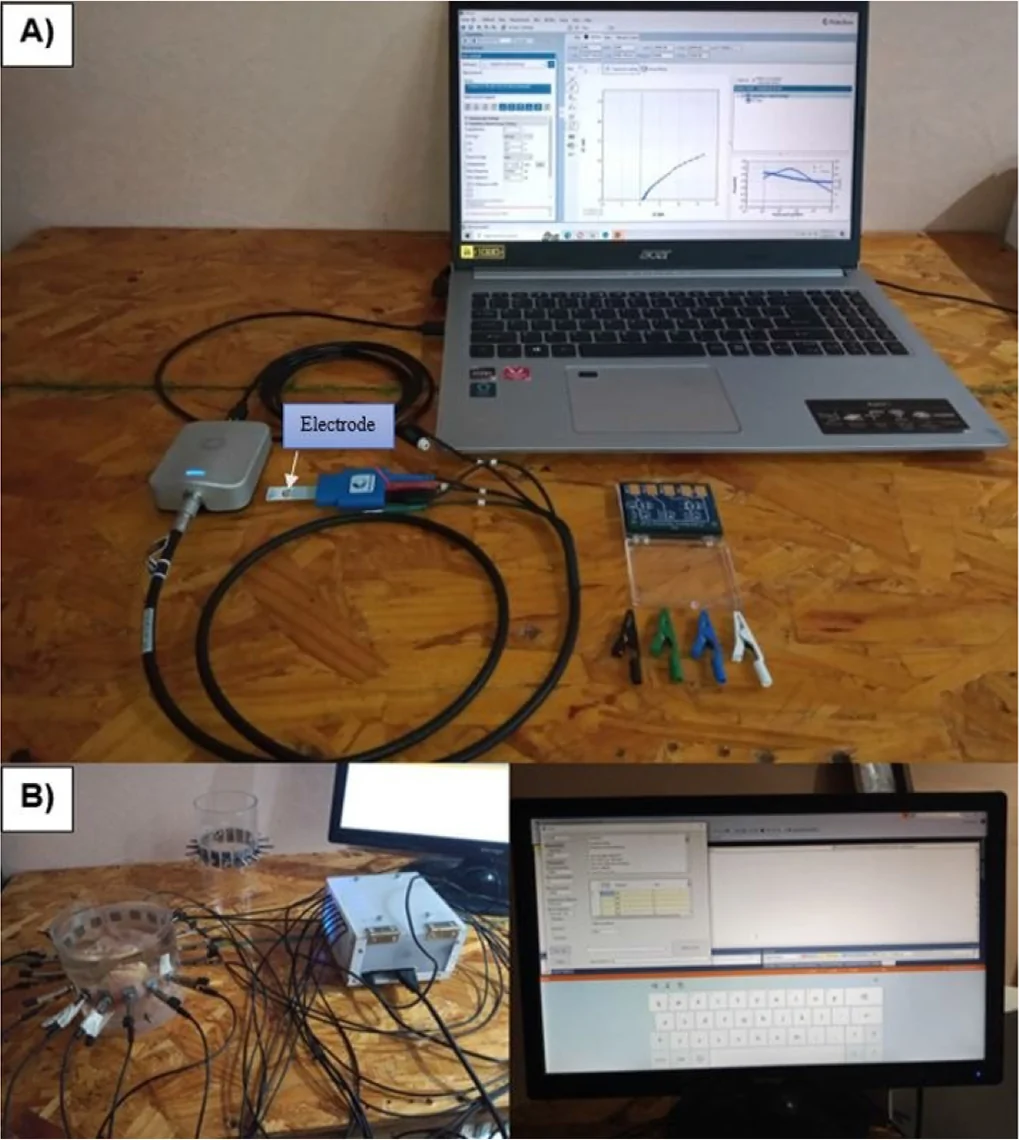
Experimental setup. A) PalmSens EmStat4s^®^, B) SBC LattePanda Alpha^®^ integrated to Arduino Leonardo^®^ board.

The measurement was conducted using one liter of water for each tissue. The sample was then introduced separately into the phantom under immersed hydration conditions using fresh tissue. The electrical contact was established by placing the electrodes in direct contact with the phantom water. Three doses of the drug were administered vertically into the center of the tissue at 2-minute intervals per trial to a depth of 2 cm. Each dose had a volume of 5 ml, resulting in a total of 15 ml per sample. The trial was repeated twice to improve accuracy and minimize measurement error. The experimental parameters are presented in Table 1.

**Table 1.** Experimental parameters for EIS and EIT analysis of biological tissues.

**Parameter**	**Beef tissue**	**Chicken tissue**	**Pork tissue**
Tissue mass	250 g	250 g	250 g
Tissue type	Muscle	Muscle	Muscle
Tissue volume	217 cm^3^	235 cm^3^	250 cm^3^
Number of samples	2	2	2
Initial condition	Fresh	Fresh	Fresh
Drug	Amoxicillin	Amoxicillin	Amoxicillin
EIS drug volume	0.25 mL	0.25 mL	0.25 mL
Number of administrations	3	3	3
Administration interval	2 min	2 min	2 min
EIS Total drug volume	1.5 mL	1.5 mL	1.5 mL
EIS frequency range	1 Hz–10 kHz	1 Hz–10 kHz	1 Hz–10 kHz
EIS electrode	Gold	Gold	Gold
EIT frequencies	10, 50, 100 kHz	10, 50, 100 kHz	10, 50, 100 kHz
EIT electrodes	16 stainless-steel	16 stainless-steel	16 stainless-steel
EIT drug volume	5 mL	5 mL	5 mL
EIT Total drug volume	15 mL	15 mL	15 mL
EIT reconstruction	Conductivity distribution	Conductivity distribution	Conductivity distribution
Administration interval	(0, 2, 4 min)	(0, 2, 4 min)	(0, 2, 4 min)
Temperature	[25°C]	[25°C]	[25°C]

The Fortran 95 programming language was used for image reconstruction, employing 104 data and a mesh of 464 elements. MATLAB^®^ (The MathWorks, Inc., Natick, Massachusetts, USA, 2018) was then used to enhance image quality using an interpolation method. Two and three-dimensional formats were used to provide a detailed view of the reconstructed images over time.

Conductivity changes in the tissue were analyzed by calculating a gradient between two consecutive images to compare pixels of the same color and obtain the contrast of the final image.

EIS and EIT provide complementary information in this study. On the one hand, EIS was used to characterize the frequency-dependent electrical response of the tissue and drug formulation at the measurement location. EIS provides primarily frequency-domain characterization, whereas EIT was used to reconstruct spatial variations in electrical conductivity within the tissue-containing phantom and provided spatial information about electrical changes following drug administration.

### Electrochemical impedance spectroscopy

EIS is a non-invasive method used to measure the electrical properties of liquids and tissues. This technique was used to analyze the resistance and reactance of the sample to obtain information about its composition and structure. Although the resistive and capacitive properties of the tissues are influenced by conductivity and permittivity, frequency played a critical role in the electrical response [18]. Figure 5 shows the equivalent circuit that models the electrode-electrolyte interface and drug delivery in the tissue. The model includes a constant phase element (CPE) in series with a resistor, connected to a parallel RC circuit, and a Warburg impedance element in parallel with a capacitor.

**Figure 5:**
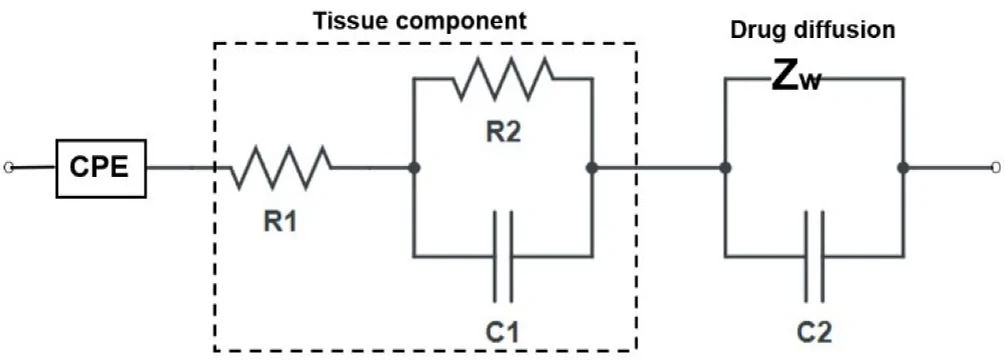
Equivalent circuit model of electrode–electrolyte interface and drug diffusion in tissue [17].

The constant phase element (CPE) is essential to the model, as it includes the physical and chemical electrode imperfections. Moreover, the Lapicque circuit represents tissue components [19]. In addition, the Warburg element in parallel with the capacitor constitutes the drug diffusion process [20], as given by Equation 1:

(1) $$Z_{\mathrm{Total}}=\frac{1}{Q{\left(j\omega \right)}^{n}}+\left[R_{1}+\frac{R_{2}}{1+j\omega R_{2}C_{1}}\right]+\frac{Z_{w}}{1+j\omega C_{2}Z_{w}}$$

where *Q* is a parameter that does not depend on the frequency, and *n* is related to the deviation from ideal capacitive behavior and phase angle. For *n* = 1, the CPE is ideal, while for lower values, the electrode shows imperfections such as surface roughness, inhomogeneity, porous structure, and the effects of the electrode-electrolyte contact. On the other hand, *R*_1_ and *R*_2_ are the resistances, *C*_1_ and *C*_2_ are the capacitances, and *Zw* the Warburg impedance used to model the diffusion process [21].

### Electrical impedance tomography

EIT is a noninvasive technique used to map the electrical conductivity of tissues [22] for image reconstruction. This method is very promising for a variety of applications, including monitoring drug propagation in ex vivo tissues [23]. The technique involves injecting current at an electrode pair and measuring the resulting potential response in the domain to determine conductivity or resistivity variations in the medium [24]. EIT allows the identification of resistivity variations in tissues owing to the color contrast in the images. To ensure that transient effects were negligible, a steady-state condition was considered, focusing on the potential distribution, composed of the umpteenth electrode *e*_1_, *e*_2_, … , *e_n_*, placed on the boundary ∂Ω, modeled by Equation 2:

(2) ∇⋅(σ∇ϕ)=0

where ∇ is the gradient, *σ* is the conductivity distribution, and *ϕ* is the electrical potential with boundary conditions given in Equation 3:

(3) $$\sigma \frac{\partial \phi }{\partial \hat{n}}=J_{n}\;\;\;\;\;\;\;\;\;\text{and}\;\;\;\;\;\;\;\;\;\sigma \frac{\partial \phi }{\partial \hat{n}}=0$$

The Neumann boundary condition represents the current flow at the boundary, where current density on the electrode surface is the normal vector. When solving the Laplace equation, both forward and inverse problems must be addressed [25]. Although the forward problem typically has a solution, the inverse problem is nonlinear and ill-posed; thus, the solution may not be unique and may involve many unknowns [26]. Consequently, small variations in the measurements or the presence of noise can cause errors or instabilities in the reconstructed image, reducing the reliability of the conductivity distribution.

### Finite element method

The finite element method (FEM) was implemented to solve the forward problem associated with the EIT measurements to obtain the spatial distribution of electrical conductivity in the tissue domain. In this study, FEM was not used only as a numerical formulation but rather as it was specifically applied to discretizing the domain that represents the experimental phantom containing the biological tissue and the 16 boundary electrodes used for electrical excitation and voltage measurement.

The circular experimental domain was discretized into two-dimensional triangular finite elements. The mesh consisted of 464 elements and was used to approximate the electrical potential distribution inside the tissue. The 16 stainless-steel electrodes were represented on the boundary of the computational domain, consistent with the electrode arrangement of the experimental EIT system. The measured electrode voltages were used as the experimental data associated with the boundary conditions of the numerical model for the reconstruction images.

For a conductivity distribution ***σ***, the electrical potential ***ϕ*** inside the domain ***Ω*** was obtained from the steady-state current conservation (Equation 2). At the electrode boundaries, the current injection and voltage measurement conditions were incorporated into the FEM formulation. For electrode ***l***, the corresponding boundary condition was expressed in Equation 3, which was then used to transform the continuous equation into a system of algebraic equations. The implemented electrode model, [27] is expressed by Equation 4:

(4) $$\int_{\Omega }\sigma \nabla \phi \cdot \nabla v\;dx+\sum_{l=1}^{n}\int_{e_{l}}\frac{1}{z_{l}}\phi v\;ds=\sum_{l=1}^{n}\int_{e_{l}}\frac{1}{z_{l}}U_{l}v\;ds$$

where ***ν*** is the mathematical weighting function, ***z_l_*** the contact impedance of the electrode, and ***U_l_*** is the measured voltage at electrode ***l***. The number of elements varies according to the problem complexity, precision, physics involved, and computational resources [28]. In this study, two-dimensional triangular elements were used to obtain the potential at each node representing the pixels in the reconstructed image. This approach allows potential dispersion patterns to be inferred on conductivity contrasts. The FEM solution was subsequently related to the EIT reconstruction procedure. Specifically, the experimentally measured boundary voltages were compared with the voltages predicted by the numerical model. Changes in the measured voltages produced corresponding changes in the estimated conductivity distribution within the finite-element domain. Consequently, regions in which the reconstructed conductivity changed after drug administration were interpreted as electrical changes associated with the spatial delivery of the drug within the tissue.

A total of 104 measurement data were used in the reconstruction. The conductivity values associated with the finite-element nodes were converted into two-dimensional images. MATLAB was subsequently used to interpolate the reconstructed data and improve the visualization of the conductivity distribution. Consecutive reconstructed images were compared by calculating conductivity gradients between successive measurements. These gradients were used to identify regions that exhibited the greatest electrical changes following drug injection. Therefore, conductivity variations were used as electrical indicators of the temporal and spatial dispersion of the administered drug, rather than as direct measurements of molecular concentration or diffusion.

### Ethical approval

This study used commercially obtained ex vivo beef, chicken, and pork tissues as experimental biological matrices. No human participants, human tissues, or live animals were involved in the experiments; therefore, ethical approval was not applicable.

## Results

### Electrical impedance spectroscopy

Frequency played an important role in determining the capacitive and resistive properties of the tissues. The capacitive elements dominated at low frequencies, and resistive effects become more pronounced at high frequencies. In particular, at 1 Hz, beef showed an impedance of 2×10^4^ Ω, pork 1.1×10^4^ Ω, and chicken 1.0×10^4^ Ω. Furthermore, drug administration resulted in a decrease in mean impedance in chicken tissue, whereas beef and pork tissues exhibited increases across the frequency range. Specifically, beef tissue decreased by approximately 50% at 1 Hz, while chicken decreased between 40 and 45% and pork by 10–15%. However, at high frequencies, the differences between tissue without the drug and tissue with the drug diminished, and the impedance values converged. The variation between control and drug-treated samples was more evident at low frequencies, as the impedance responses converged at high frequencies. Thus, the behavior of the electrical response can be associated with drug administration depending on frequency. However, additional experimental validation is required to determine the specific contributions of extracellular and intracellular pathways, which cannot be determined solely from the impedance curves. Regarding the phase angle, it was negative across the frequency range, owing to the probable presence of reactive electrical components in the measured samples. For low and intermediate frequencies, pork showed phase angles of −35° to −52°, whereas the response values were higher between −5° and −15°. The results indicate changes that depend on the frequency, reactive behavior of the tissues, and the drug formulation (Figure 6).

**Figure 6:**
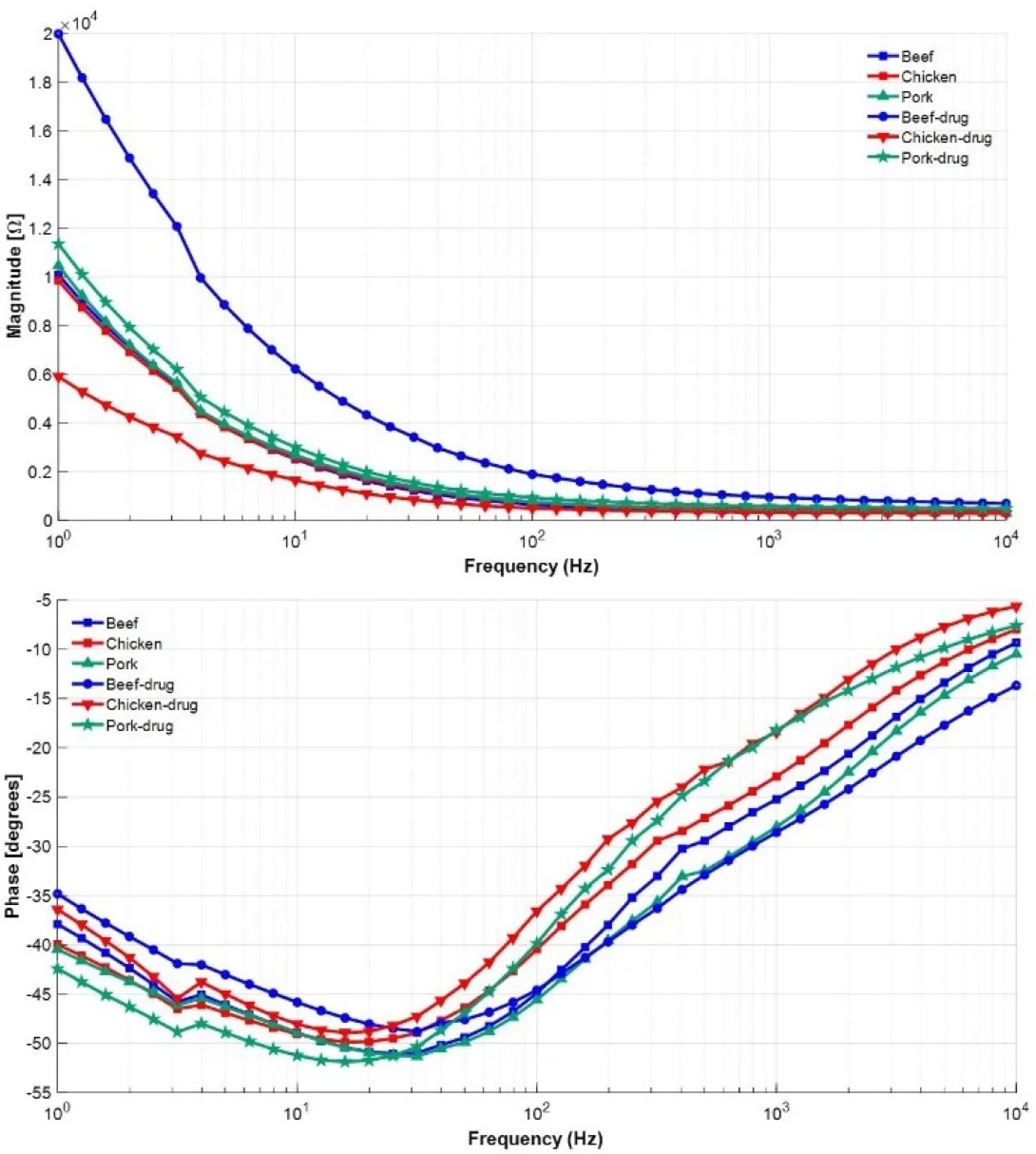
Summary for drug and tissue state-space fitting model impedance data in the frequency range 1 Hz to 10 kHz.

The system identification was used to characterize the resistive and capacitive behavior related to the electrochemical responses, following the frequency-domain system identification approach described in [29]. To obtain the best model, an algorithm in MATLAB was developed to determine the approximately optimal transfer function. A second-order transfer function with two poles and two zeros was considered to model the experimental impedance. The model was evaluated by comparing the estimated frequency response with the experimental data. The fit accuracy was above 90% for all samples, beef (92.25), chicken (92.82%), pork (92.49%), and drug (92.34%), with beef exhibiting the lowest value. Therefore, the results show that the selected model provides a good approximation of the measured response.

The RC components of the equivalent circuit for tissues and drug were calculated in the same way for all samples listed in Table 2.

**Table 2.** Summary of the equivalent circuit model with two poles and two zeros using systems identification.

**%Fit**	**Beef**	**Chicken**	**Pork**	**Drug**

	92.25%	92.82%	92.49%	92.34%
R_1_ (Ω)	386.24	502.0	342.79	571.47
R_2_ (Ω)	2071.49	2274.92	1691.52	1984.57
R_3_ (Ω)	1.18×10^4^	1.31×10^4^	6.49×10^3^	1.05×10^4^
C_2_ (F)	3.5×10^−5^	3.6×103.5×10^−5^	3.3×103.5×10^−5^	3.3×103.5×10^−5^
C_3_ (F)	8.0×103.5×10^−5^	8.1×103.5×10^−5^	1.0×103.5×10^−4^	1.1×103.5×10^−4^

where R_1_ is associated with the extracellular medium, R_2_ with internal tissue resistance, and R_3_ with the resistive behavior of intracellular interactions [30]. Furthermore, C_2_ models the double layer at the electrode-tissue interface, and C_3_ models tissue polarization. In particular, pork with drug exceeded 10^−4^ F, which may be due to probable polarization or capacitive effects [31]. Moreover, chicken showed high resistance, which may be due to its anisotropic muscle structure and compositional differences rather than water content [32].

Figure 7 shows normalized differences in R_1_, R_2_, R_3_, C_2_, and C_3_ with respect to the maximum value of each parameter to eliminate the scale effects of the resistive and capacitive elements. The electrical impedance responses exhibited variations in resistive and reactive properties over the frequency range. The differences in the parameters revealed resistive and capacitive components among the tissue samples and the drug formulation, with variations specifically in R_3_ and C_3_ of the samples. These parameters may be useful for distinguishing their electrical responses, as they show variations in charge transport and polarization.

**Figure 7:**
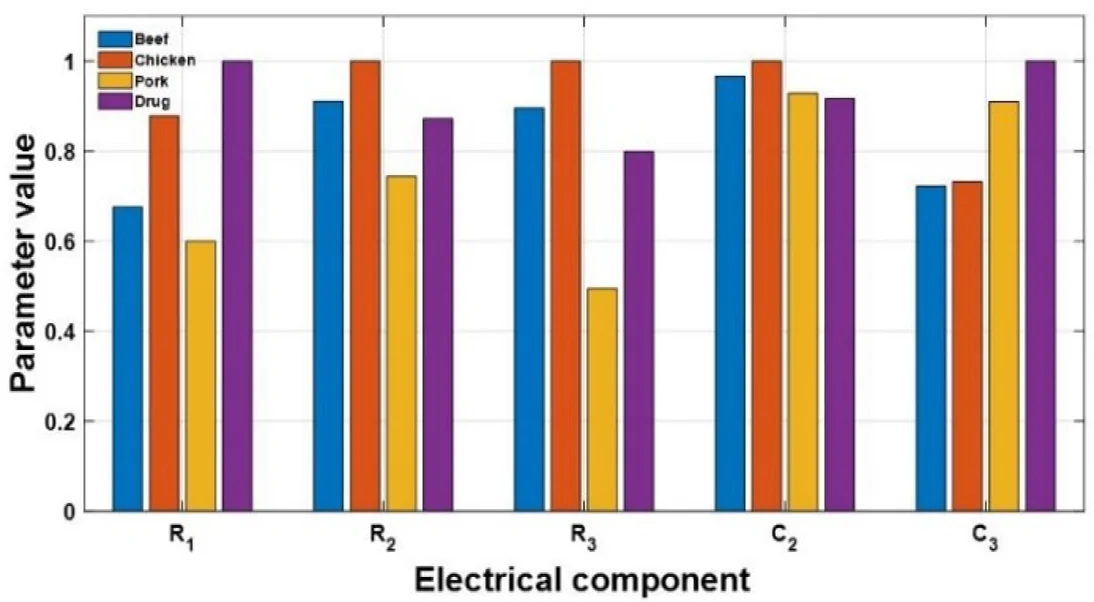
Normalized Differences Between Samples.

However, a limitation of the equivalent circuit is that it cannot be regarded as a unique indicator of specific physical or chemical processes, because different circuit configureations may produce similar impedance responses. Moreover, resistance and capacitance values may be influenced by tissue heterogeneity, moisture content, and measurement conditions. Therefore, changes in parameters following amoxicillin administration should be interpreted as changes in the overall electrical response of the tissue, not as direct evidence of a specific drug transport mechanism.

The state-space model was identified as a viable representation to determine the component-associated electrochemical process to link the physical and electrochemical phenomena [33]. The procedure involved analyzing the transfer functions of each sample. To demonstrate the process, the beef model was selected in the frequency range of 1–10 kHz, as shown in Equation 5:

(5) $$G{\left(s\right)}_{\mathrm{Beef}}=\frac{388.4s^{2}+4.66\times 10^{4}s+2.07\times 10^{5}}{s^{2}+14.81s+14.57}$$

At low frequencies, the DC gain shows a steady-state behavior in the system due to constant or slowly varying inputs. However, as the frequency increases, the dynamics are influenced by the poles and zeros that modify the amplitude and phase response, as they mark off stability and transient behavior, as described in Equation 6:

(6) $$G_{DC\mathrm{Beef}}=K\cdot \frac{\left(-z_{1}\right)\left(-z_{2}\right)\ldots \left(-z_{m}\right)}{\left(-p_{1}\right)\left(-p_{2}\right)\ldots \left(-p_{n}\right)}$$

where *K* ≠ *G*(0) unless all zeros and poles are located at the origin or cancel proportionally. Each factor influences the steady-state gain, and it establishes the reference response before the dynamic effects of poles and zeros become significant, thus yielding the DC gain denoted by Equation 7:

(7) $$\underset{s\to 0}{\lim}G{\left(s\right)}_{\mathrm{Beef}}=\frac{2.072\times 10^{5}}{14.57}\approx 14217.43$$

where *s* = *jω*. As frequency increases, the lower-order terms in the numerator and denominator become negligible, and the system is dominated by the higher order coefficients, as described in Equation 8:

(8) $$G{\left(j\omega \right)}_{DC\mathrm{Beef}}=K\ldots \frac{\left(j\omega -Z_{1}\right)\left(j\omega -Z_{2}\right)\ldots \left(j\omega -Z_{m}\right)}{\left(j\omega -p_{1}\right)\left(j\omega -p_{2}\right)\cdot \left(j\omega -p_{n}\right)}\approx \left|K\right|\cdot \omega^{m-n}$$

At very high frequencies, the gain approaches a constant value determined by the highest-order terms; thus, in the limit as *s* → ∞, Equation 9 is obtained.

(9) $$\underset{s\to \infty }{\lim}G{\left(s\right)}_{\mathrm{Beef}}\approx 3.884$$

The poles and zeros influence the system response in both the time and frequency domains. Figure 8 presents a Bode plot for the beef tissue, which provides information on the individual contributions of the poles and zeros relative to the DC gain. It also shows the changes in magnitude and phase dynamics at low frequencies for the steady-state system.

**Figure 8:**
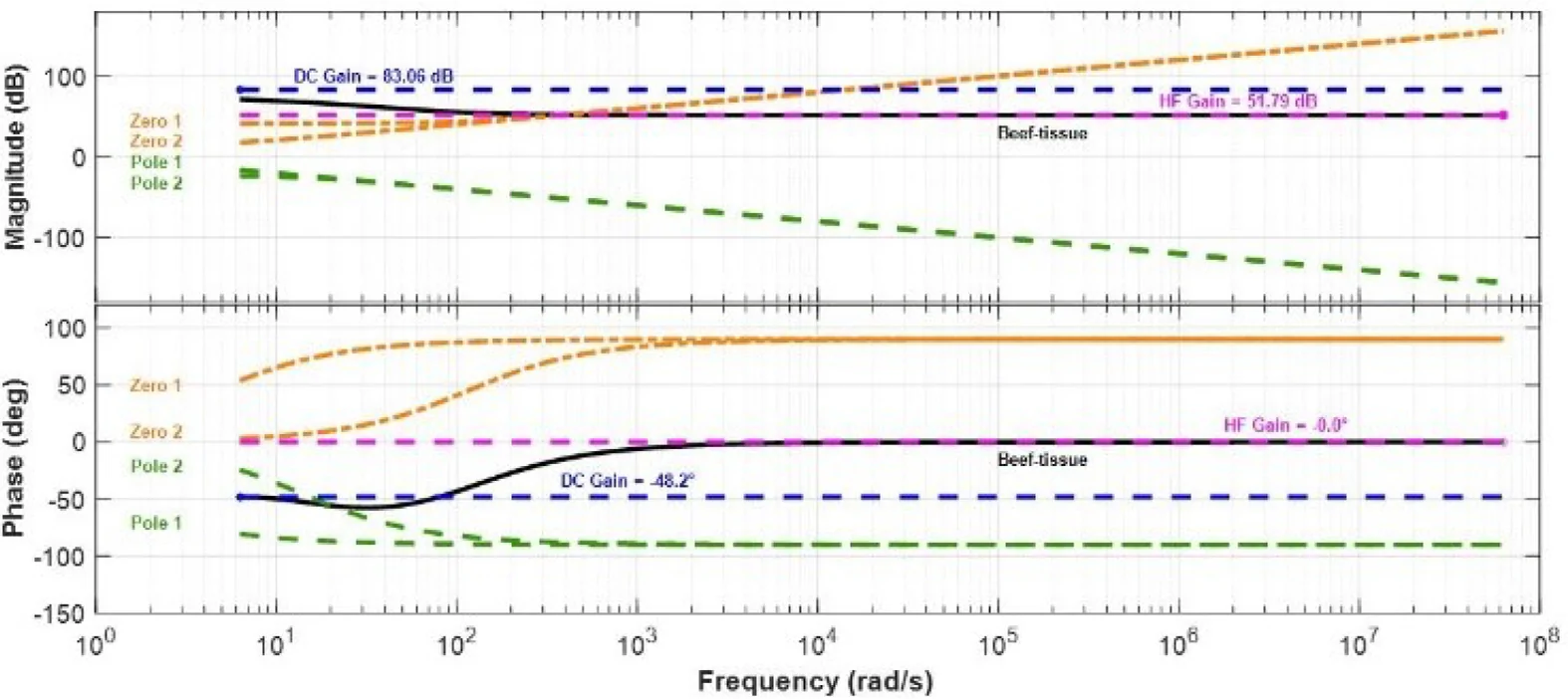
Bode plot for beef tissue showing the individual contributions of each pole and zero.

### Electrochemical impedance

The electrical behavior of materials depends on structure and composition. Some factors that varied in measurement were fat and water content, which may influence ionic mobility and charge transport mechanisms. Another factor was the frequency, which is directly related to the electrical response, as current travels through extracellular and intracellular regions. Particularly the beef showed higher conductivity compared to the other tissues at medium and high frequencies, where the resistive and capacitive components formed an arc that models the electrochemical RC system using a simple Randles circuit [21]. In contrast, the drug showed a low impedance and a diffusion potential associated with dispersion in the cellular regions of the tissue, where ionic mobility is high and resistive opposition to AC signals is low. In a simplified representation Nyquist diagrams exhibited differences in impedance behavior among the tissue and the drug. The responses were modeled using equivalent circuit components, including a CPE [34, 35, 36] and Warburg component to provide phenomenological representations of non-ideal interfacial behavior and diffusion related to the impedance contributions (Figure 9) [37, 38].

**Figure 9:**
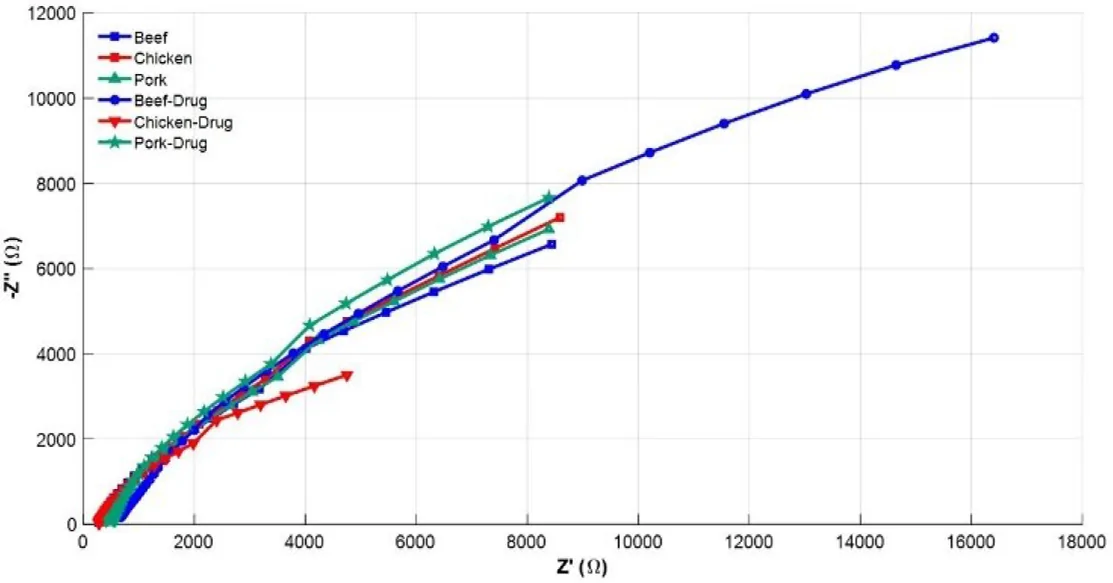
Nyquist diagrams for tissues and drug.

A CPE and Warburg impedance components require rigorous mathematical analysis; however, this work focuses solely on estimating linear models to obtain an equivalent circuit [39]. Therefore, the constant phase elements are approximations that represent the network of passive components in state-space models used to describe the dynamic behavior of the system [29, 40, 41].

To evaluate the effect of the drug on the tissue types, a sample size of 41 was used. The paired-samples t-test was conducted on the pairwise differences between tissue with drug versus control. The t-test has (*df* = *n* − 1 = 40) degrees of freedom, where the comparisons were carried out across the three groups using Bonferroni correction to adjust the p-values for multiple comparisons. Moreover, descriptive and effect-size metrics for mean differences. Hedges’ g, a correction of Cohen’s d, was also computed to measure the magnitude of the differences. The paired-samples t-test was conducted with *n* = 40 per group. Beef showed an increase in mean impedance from 2027.36 ± 2643.90 (control) to 4622.99 ± 5369.22 (treated), chicken resulted in a difference of 2062.64 ± 2545.45 to 1306.68 ± 1536.33 and pork showed a slight increase of 2123.26 ± 2694.16 to 2421.42 ± 2908.58. Beef variation was statistically significant, *t*(40) = 6.07, *p* < 0.001 (Bonferroni-adjusted *p* = 1.15 × 10^−6^), with a large effect size (Cohen’s *d* = 0.93). In contrast, chicken exhibited a reduction in impedance after drug application, with a decrease in mean value, corresponding to a difference of −755.96 (95% CI: −1074.75 to −437.16) and a variation of *t*(40) = −4.79, *p* < 0.001 (Bonferroni-adjusted *p* = 6.86 × 10^−5^), with a moderate to large effect size (Cohen’s *d* = −0.73). Finally, pork showed an increase in impedance values, with a mean difference of 298.16 (95% CI: 229.47 to 366.85), *t*(40) = 8.77, *p* < 0.001 (Bonferroni-adjusted *p* = 2.19 × 10^−10^), and Cohen’s *d* = 1.34. Table 3 presents the statistical summary.

**Table 3.** Comparison between control and drug-treated tissue across the impedance spectrum.

**Tissue**	**Beef**	**Chicken**	**Pork**
Mean control	2027.36	2062.64	2123.25
SD control	2643.90	2545.45	2694.16
Mean treated	4622.99	1306.68	2421.41
SD treated	5369.22	1536.33	2908.57
Mean difference	2595.63	−755.96	298.16
Lower limit at 95%	1730.71	−1074.75	229.47
Upper limit at 95%	3460.56	−437.16	366.85
t-value	6.07	−4.79	8.77
Degrees of freedom	40	40	40
p-value	3.82×10^−7^	2.28×10^−5^	7.31×10^−11^
p-Bonferroni	1.14×10^−6^	6.86×10^−5^	2.19×10^−10^
Cohen-d	0.93	−0.73	1.34

Paired t-test with Bonferroni correction applied.

## Discussion

To assess drug propagation, vector maps that included impedance gradients were used, with vectors converging toward the region of maximum conductivity in a defined pattern, suggesting progressive spatial propagation [42].

Although the images show noise at the interpolated points, probably due to the interaction between the injected current and the measurement electrode pair, they still display resistivity changes in the study domain [43]. Figure 10A shows consecutive reconstructions of beef tissue. The conductivity distribution extends from the periphery toward the central region. Moreover, high conductivity areas present spatial variations in the electrical response to drug administration. These variations may be associated with changes in the local ionic environment and the administered solution. On the other hand, Figure 10B provides conductivity patterns, highlighting their spatial evolution over time.

**Figure 10:**
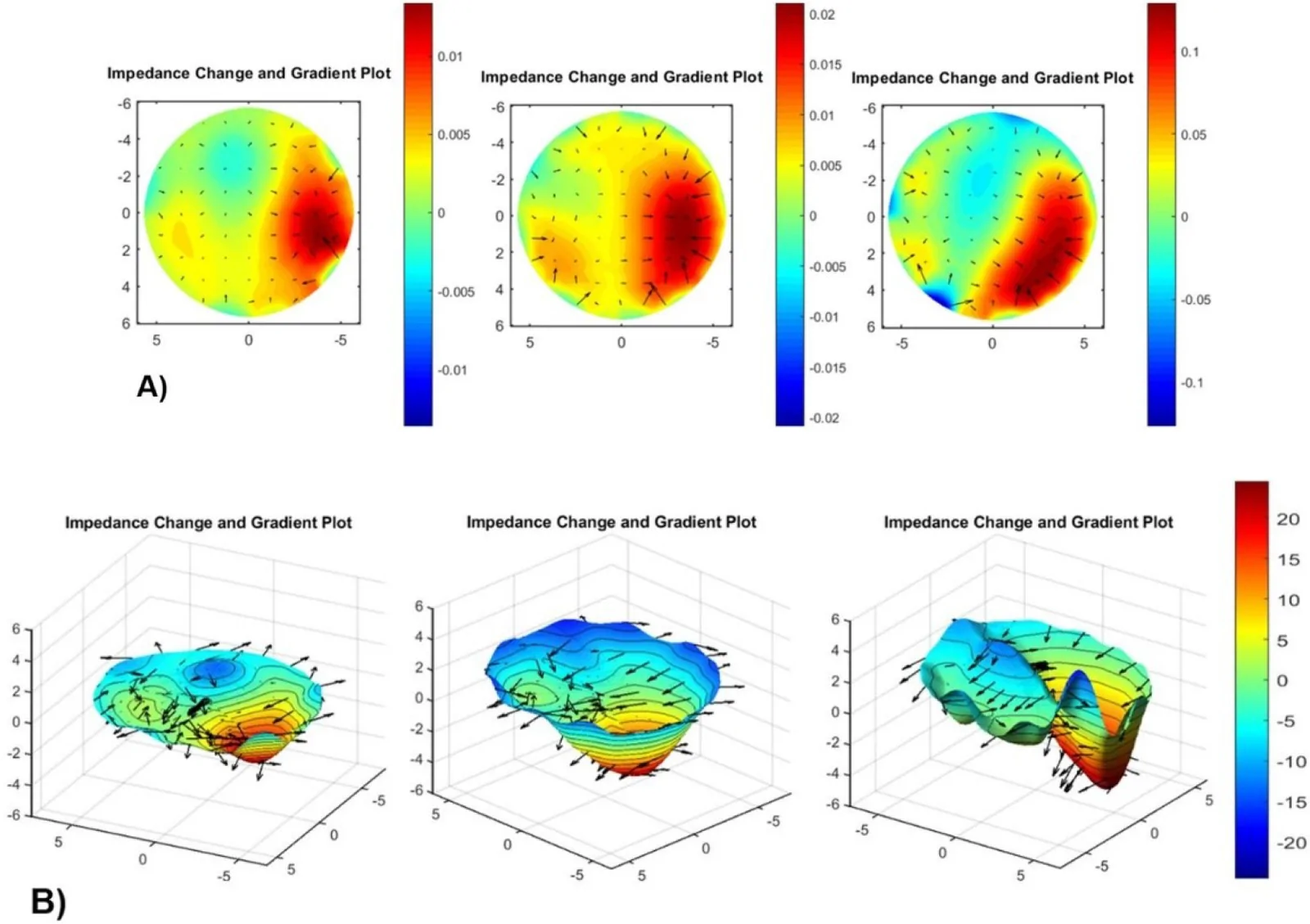
Spatial conductivity changes associated with drug administration in beef tissue. A) two-dimensional and B) three-dimensional for 400 mV and 50 kHz.

On the other hand, Figure 11A presents homogeneous conductivity distribution, characterized by weaker spatial gradients and smaller variations in the study domain. The vectors indicate the spatial orientation of electrical variations. Figure 11B provides the conductivity patterns, illustrating their spatial distribution in the tissue. Although the observations suggest a different electrical response in beef tissue, they do not directly establish the direction or rate of drug diffusion, as differences in tissue composition, hydration, and structural heterogeneity may also contribute to the conductivity patterns [44].

**Figure 11:**
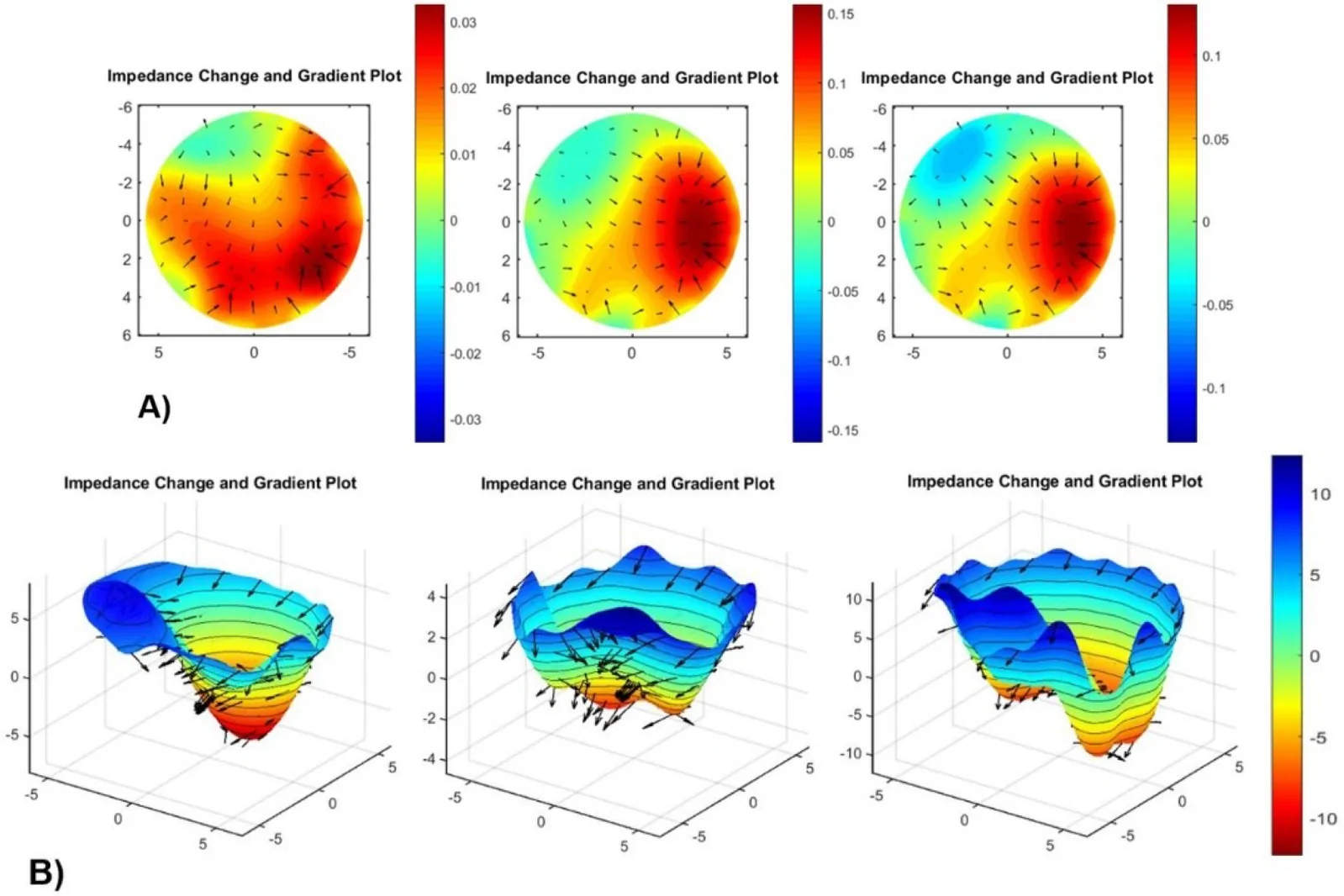
Spatial conductivity changes associated with drug administration in chicken tissue. A) two-dimensional and B) three-dimensional for 400 mV and 10 kHz.

The conductivity changes in pork tissue. Figures 12A and 12B show an approximately radial distribution of the conductivity variations in pork tissue. The uniform spatial pattern and limited changes between consecutive reconstructions suggest an apparent stabilization of the electrical response during the observation period. However, the spatial symmetry and temporal variations are insufficient to establish that the drug reached physicochemical equilibrium. Additional quantitative temporal analysis and independent chemical measurements would be necessary to confirm the drug distribution and determine its diffusion behavior within the tissue. Moreover, the electrical variations should not be interpreted as direct measurements of drug concentration or molecular diffusion.

**Figure 12:**
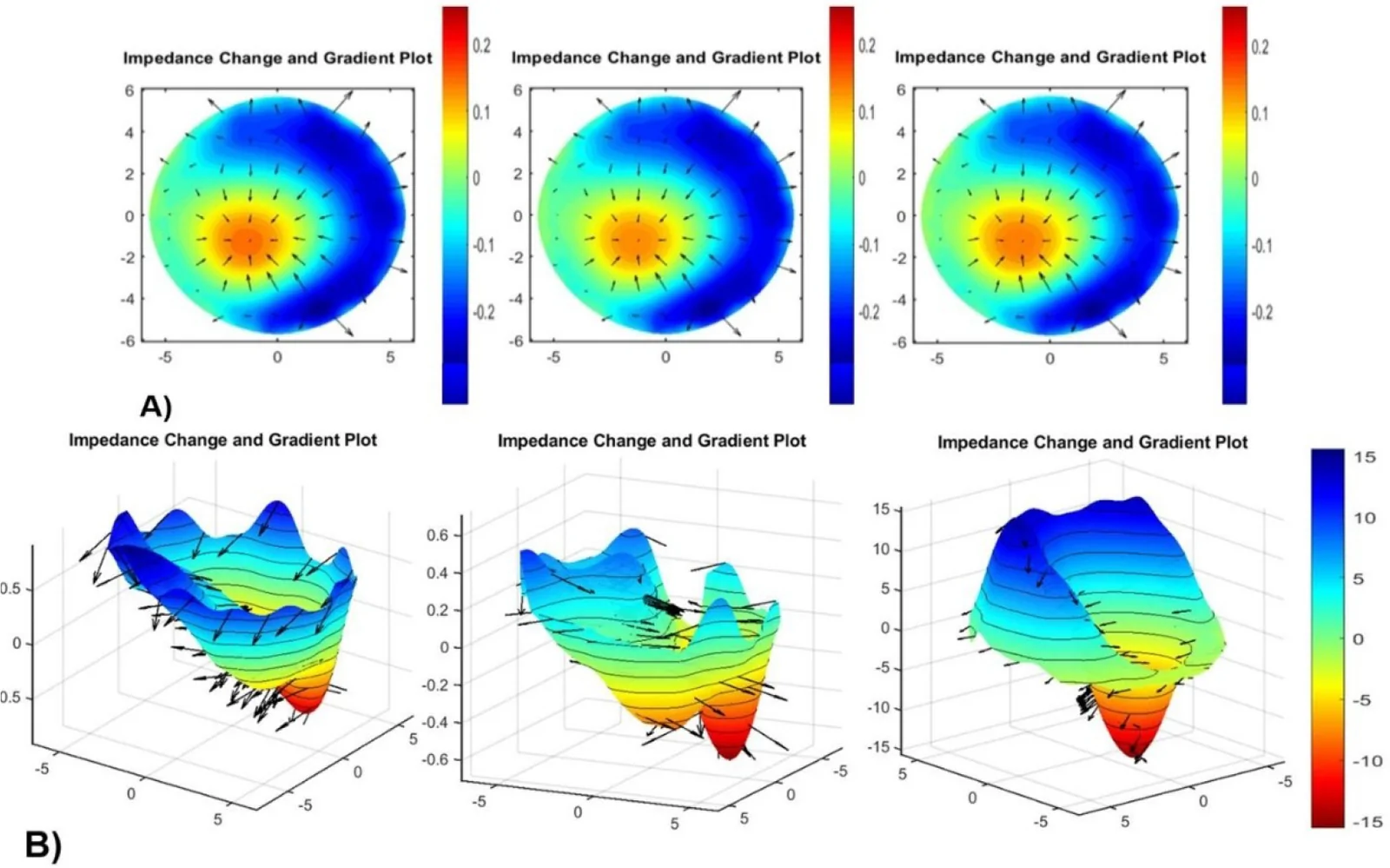
Spatial conductivity changes associated with drug administration in pork tissue. A) two-dimensional and B) three-dimensional for 200 mV and 50 kHz.

To validate drug dispersion in tissues, an additional measurement in the beef tissue was performed over 0–22 minutes. Consecutive images show the temporal evolution of the impedance distribution in the tissue, with changes in both the spatial pattern and the magnitude of the electrical properties (Figure 13A). All impedance changes were calculated relative to the baseline measurement. In addition, positive and negative changes in magnitude identify areas where the electrical response differs from the initial state. In fact, localized variations indicate spatially heterogeneous responses, whereas widespread changes suggest that the variation extends across a larger portion of the reconstructed domain (Figure 13B). Thus, the image sequence highlights regions where impedance increases compared to the initial image. However, localized irregularities appear at the boundary, and spatial variations are observed in the measurements. Therefore, the pattern suggests that the area exceeding the threshold changes rapidly during the initial stage and then stabilizes over time (Figure 13C).

**Figure 13:**
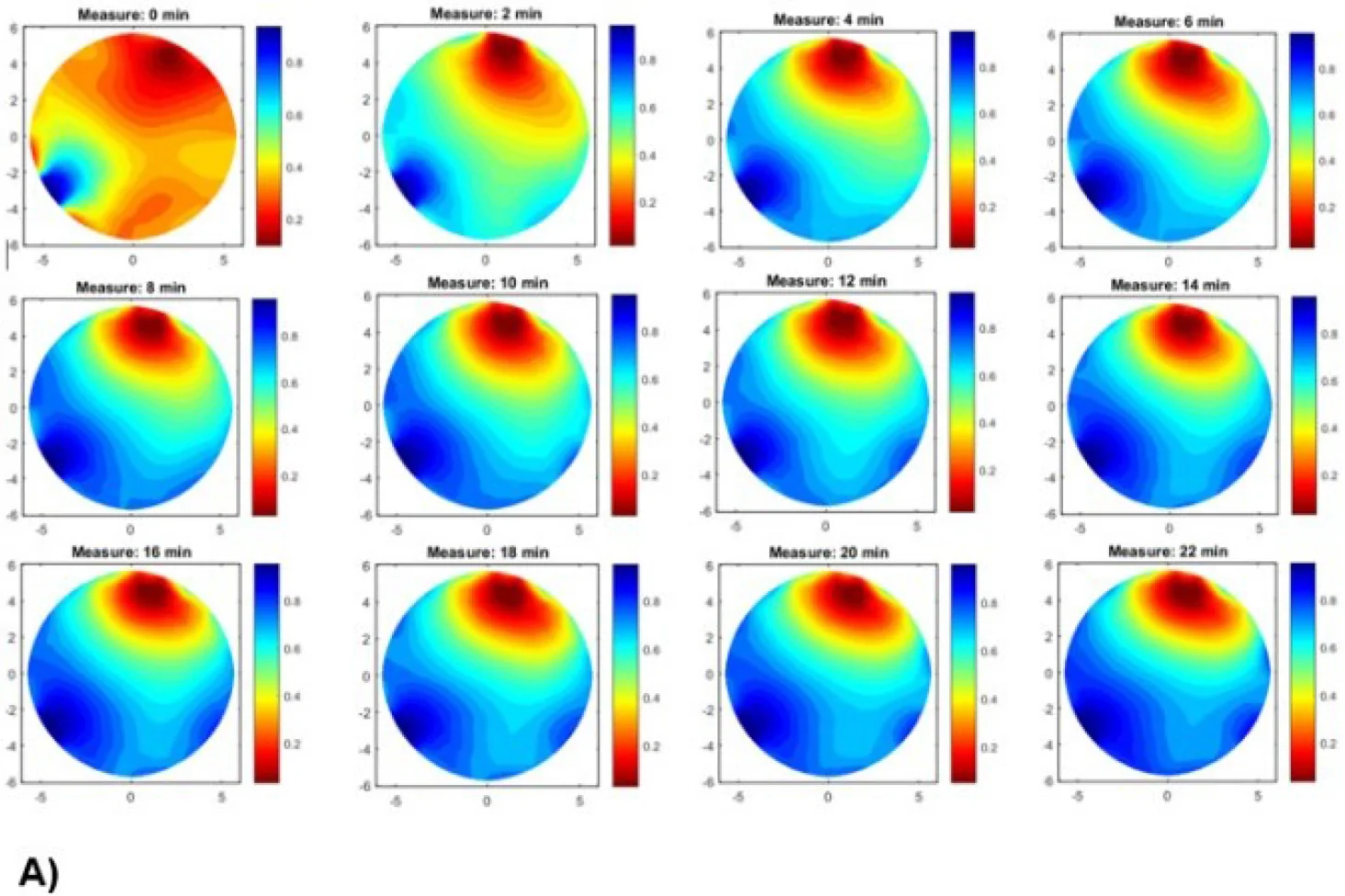

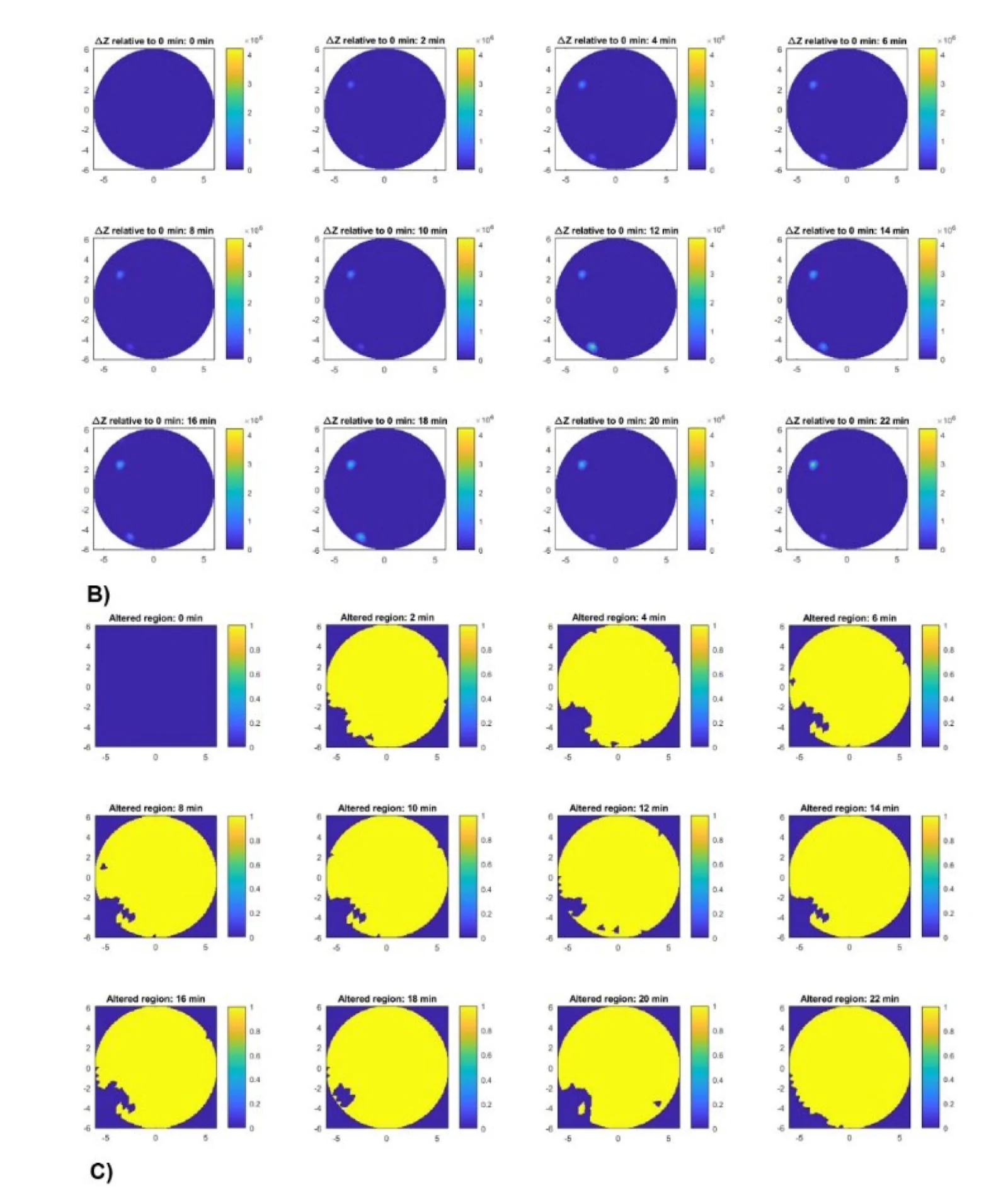
A) EIT image reconstruction in beef tissue at different times. B) Spatial distribution of impedance changes relative to baseline. C) Region with impedance change.

The area of drug delivery in the tissue increased rapidly during the initial interval, reaching approximately 95% at 2 minutes, and remained roughly stable throughout the subsequent measurements (Figure 14A). In contrast, the centroid coordinates showed temporal variability and continuous spatial redistribution within the affected region (Figure 14B). The root mean square (RMS), however, remained within a narrow range, suggesting relatively constant centroids despite changes in centroid position (Figure 14C). The most pronounced centroid displacement occurred between 16 and 18 minutes, followed by a partial reversal at 20 minutes (Figure 14D). Thus, the spatial position varied more markedly than the overall affected area or RMS dispersion radius during the observation period.

**Figure 14:**
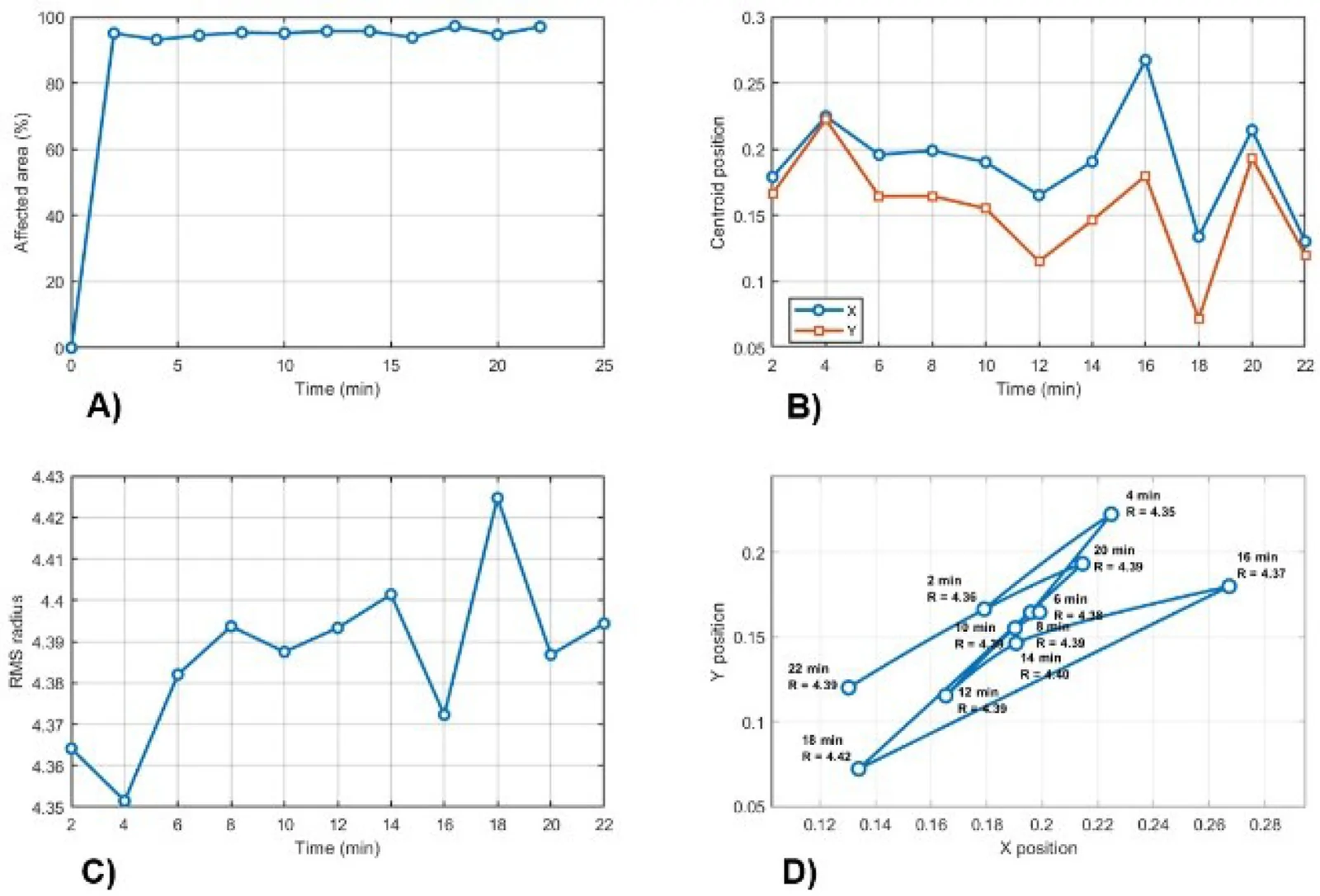
Summary for A) temporal evolution of spatial extent, B) centroid movement, C) dispersion radius, and D) centroid trajectory and dispersion.

Table 4 presents the quantitative results that describe the temporal evolution of the affected region by the drug in the tissue over a 22-minute observation period.

**Table 4.** Summary of quantitative results.

**Time (min)**	**Points**	**Area (%)**	**Centroi d X**	**Centroi d Y**	**RMS**
0.0	0.0	0.0	NaN	NaN	NaN
2.0	441	95.04	0.179	0.166	4.36
4.0	432	93.10	0.225	0.222	4.35
6.0	438	94.40	0.196	0.164	4.38
8.0	442	95.26	0.199	0.165	4.39
10.0	441	95.04	0.190	0.155	4.38
12.0	444	95.69	0.165	0.115	4.39
14.0	444	95.69	0.191	0.146	4.40
16.0	435	93.75	0.267	0.180	4.37
18	451	97.20	0.134	0.072	4.42
20.0	439	94.61	0.214	0.193	4.38
22.0	450	96.98	0.130	0.120	4.39

An important factor to consider is that structural differences in the tissues, such as hydration and porosity, could influence the electrical response and the spatial distribution of the injected drug [45]. Although all tissues were measured under equal hydration conditions, intrinsic characteristics influenced each sample, probably affecting ionic transport, local conductivity, and the resulting impedance. Nevertheless, the measurements do not independently quantify water-mediated transport or molecular diffusion rates. Thus, the observed differences among beef, chicken, and pork should be interpreted as tissue-dependent electrical responses associated with drug administration. In particular, these results are notable for structural and physiological similarities to human tissues, making them valuable models for predicting drug behavior in preclinical studies [46].

To quantify the spatial resolution and localization performance of the EIT reconstruction, the parameter FWHM was used. Accuracy was evaluated by comparing the reconstructed peak position with the injection site. The analysis revealed high reconstruction reliability near the electrode regions and decreased sensitivity in the central domain, consistent with the observed spatial smoothing [47, 48]. Table 5 summarizes the spatial resolution values.

**Table 5.** Quantitative spatial resolution values for the reconstructed images.

**Tissue**	**FWHM (pixels)**	**Localization error (pixels)**	**Relative error (error/FWHM)**
Beef	261.57	156.31	0.59
Chicken	262.23	116.28	0.44
Pork	299.69	145.34	0.51

The results in Table 5 show drug dispersion in beef. However, propagation is more localized in chicken tissue, where the conductive gradients are most well-defined. In contrast, pork exhibits high dispersion due to its porous structure, which promotes greater interstitial diffusion and reduces impedance contrast. Although the FWHM values ensure reproducibility of the measurement, there is a percentage error relative to the resolution of the reconstructed images (Figure 15).

**Figure 15:**
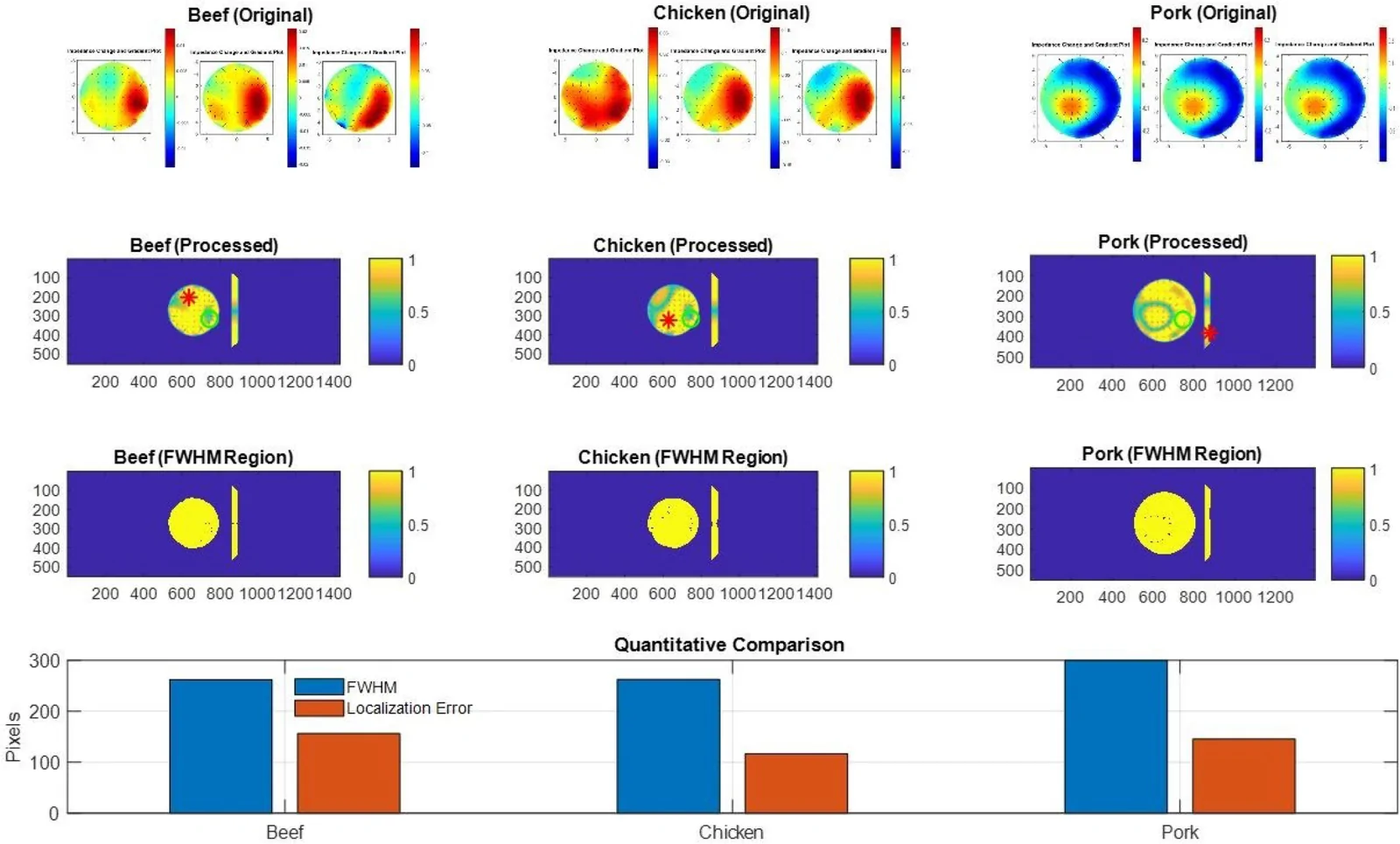
Comparison of EIT reconstructions and spatial resolution metrics: FWHM and localization error.

The localization error is approximately 44 to 59% relative to FWHM, which is a typical value in image reconstruction for EIT due to its ill-posed nature [49]. However, the spatial resolution analysis revealed similar FWHM values for beef and chicken, approximately 262 pixels, whereas pork presented a major value of 300 pixels. The localization error was 156 pixels for beef, 116 pixels for chicken, and 145 pixels for pork. These values are consistent with the relative error of the ranges between 0.44 and 0.59. Furthermore, the large FWHM value for pork may be interpreted as a broader reconstructed spatial response, but it cannot be evidence of greater molecular diffusion.

## Conclusion

EIS and EIT were used to characterize electrical changes induced by the administration of amoxicillin in ex vivo tissues. EIS measurements presented tissue-dependent impedance responses across the frequency range, with differences between control and drug-treated samples. EIT reconstructions provided spatial information about conductivity variations following drug administration. However, the patterns differed among tissues with variations in the electrical response and in the spatial distribution of the injected tissue. The FWHM and localization error provided quantitative information about the spatial resolution and localization of reconstructions. Tissue heterogeneity, together with the characteristics of the drug, induced changes in diffusion, modifying stability, solubility, and the delivery pathway. The conductivity changes were analyzed using gradients across consecutive images, highlighted by color contrast. The regions of high conductivity were designated as drug distribution patterns in the biological samples. An important factor was related to structural differences between chicken and beef; both showed similar conductive properties. However, drug dispersion was more extensive in beef than in chicken, probably due to differences in density, fiber arrangement, and porosity. In contrast, porcine tissue showed circular diffusion of the drug toward the center of the tissue. This pattern may be attributed to the high fat content, which reduces liquid dynamics and increases viscosity, resulting in slower convection during diffusion processes. To support the current study, an additional measurement of drug propagation in beef tissue was proposed over 0–22 minutes, specifically showing drug dispersion in the tissue as a function of impedance variation. Nevertheless, further studies are necessary to incorporate independent chemical measurements to establish a direct relationship between conductivity variations and drug distribution.

Future work will focus on the dispersion analysis of chemotherapy agents administered in porcine tissue using the locoregional method to compare the pharmaceutical drug diffusion behavior.
